# Circulating and disseminated tumor cells: diagnostic tools and therapeutic targets in motion

**DOI:** 10.18632/oncotarget.12242

**Published:** 2016-09-24

**Authors:** Hongxia Wang, Nikolas H. Stoecklein, Peter P. Lin, Olivier Gires

**Affiliations:** ^1^ Department of Oncology, Shanghai General Hospital, Shanghai Jiao Tong University School of Medicine, Shanghai, P.R. China; ^2^ Department of General, Visceral and Pediatric Surgery, Medical Faculty, University Hospital of the Heinrich-Heine-University Düsseldorf, Düsseldorf, Germany; ^3^ Cytelligen, San Diego, California, USA; ^4^ Department of Otorhinolaryngology, Head and Neck Surgery, Grosshadern Medical Center, Ludwig-Maximilians-University of Munich, Munich, Germany; ^5^ Clinical Cooperation Group Personalized Radiotherapy of Head and Neck Tumors, Helmholtz, Germany

**Keywords:** CTCs, DTCs, metastases, EpCAM, MICs

## Abstract

Enumeration of circulating tumor cells (CTCs) in peripheral blood with the gold standard CellSearch^TM^ has proven prognostic value for tumor recurrence and progression of metastatic disease. Therefore, the further molecular characterization of isolated CTCs might have clinical relevance as liquid biopsy for therapeutic decision-making and to monitor disease progression. The direct analysis of systemic cancer appears particularly important in view of the known disparity in expression of therapeutic targets as well as epithelial-to-mesenchymal transition (EMT)-based heterogeneity between primary and systemic tumor cells, which all substantially complicate monitoring and therapeutic targeting at present. Since CTCs are the potential precursor cells of metastasis, their in-depth molecular profiling should also provide a useful resource for target discovery. The present review will discuss the use of systemically spread cancer cells as liquid biopsy and focus on potential target antigens.

## INTRODUCTION

Metastasis is the major cause of cancer-related death [1]. Growing evidence supports the notion that locally invading, blood-borne circulating tumor cells (CTCs) and disseminated tumor cells (DTCs) in bone marrow and lymph nodes are precursors of recurrent tumors and metastases. So far, the development of targeted therapies was mostly fueled by knowledge related to primary tumor biology and, currently, around one dozen therapeutic antibodies and 28 different inhibitors are in clinical application, targeting essentially the tumor antigens HER2, EGFR, EpCAM, BRAF and VEGF [2–5]. These therapeutic agents have been primarily approved for late stage advanced disease with recurrent tumors and/or distant metastases [2–4]. Owing to technical and study limitations, the above mentioned therapeutics are barely in use to target (occult) precursors of recurrence and metastases in first-line therapies. In fact, currently available therapeutic agents are generally applied when cellular precursors have already deployed their capacities and disease has progressed. Nevertheless, efforts to enumerate occult systemic cancer cells and to transfer molecular therapies to earlier, less progressed stages of disease have been undertaken in breast cancer [6–9], which hopefully represents a trailblazer for other entities.

In order to change treatment regimens towards more effective suppression of metastasis, two aspects are paramount. Firstly, a more accurate staging including diagnosis of the systemic disease is mandatory, in order to define patients at increased risk to relapse and/or to develop metastases. Such diagnostics must detect clinically occult systemic cancer that is currently not assessable by routine diagnostics. In our opinion, CTCs and DTCs appear to be good candidates to achieve this aim. Secondly, reliable and validated assays for detection and molecular analysis of CTCs/DTCs are required in the therapeutic context, which is currently very challenging in the adjuvant situation. The technologies required for such molecular characterization of CTCs and DTCs should optimally enable not only assessment of known therapeutic targets, *e.g.* HER2, EGFR, and EpCAM, but also comprehensive profiling to identify novel therapeutic targets. Such molecular staging becomes even more important in the adjuvant situation in light of reported discordances in expression patterns of several therapeutic targets in primary tumors *versus* CTCs and DTCs [10–14]. It appears therefore mandatory, to determine the exact target expression in systemic cancer cells to select the correct adjuvant therapy in the non-metastatic, high-risk situation in the context of precision medicine.

In the present review, we will discuss advantages and challenges related to CTCs and DTCs as diagnostic tools and therapeutic targets in motion. We will briefly summarize knowledge on enumeration and characterization, and extend on potential molecular targets on the cells of systemic cancer.

## CTCS AS LIQUID BIOPSIES

Reliable biomarkers for molecular staging of disease progression and risk evaluation of carcinomas have, as yet, entered clinical routine only for a subset of tumor entities, such as the PSA protein in prostate cancer. In this context, CTCs could play a role as “liquid biopsy” through their direct molecular characterization to obtain comprehensive “on-line” information on the extent and the molecular phenotypes of systemic cancer [12, 15–20]. In the metastatic situation, CTCs have prognostic significance in various tumor entities (Table 1). In a large cohort of non-metastatic primary breast cancer patients (*n* = 3173), one or more CTCs were found in approximately 20% of individuals at the time of first diagnosis and strongly correlated with larger tumors, nodal involvement, and poor disease outcome [21]. Hence, CTCs are also detectable in the adjuvant, non-metastatic situation, although at reduced rates and numbers, and have prognostic impact.

**Table 1:** Current molecular markers for the identification and therapeutic targeting of CTCs and DTCs in solid cancers

Biomarker	Expression rate	Drug	Description
**Markers for CTCs**			
**EpCAM**	37-42.3%	Panorex, MT201, MT101, ING-1	FDA-approved CellSearch^TM^ system depends on EpCAM-specific capturing of CTCs in various cancers [21, 30, 47, 49, 125, 127, 258–262] Loss of EpCAM on CTCs as a result of dynamic phenotypic changes during EMT[11, 35, 150, 151, 263]
**CD44**	35.2%	Pan-CD44 antibody H90	CD44 expression in CTCs of HNSCC, breast, gastric and endometrial cancer patients [46, 264–268]
**ALDH1**	17.7-80%	ATRA, DEAB	ALDH1 expression in CTCs of breast, non-small cell lung and endometrial cancer patients [36, 264, 266, 267, 269]
**CD133**	83%	CART133 chimeric antigen receptor (CAR) T cells	Expression of the cancer stem cell marker CD133 in CTCs of metastatic breast, colon, colorectal, renal cell, hepatocellular and non-small cell lung cancer patients [266, 269–257]
**FGF2**	n.a.	Dovitinib, Pentraxin-3	Frequent secretion of FGF2 by CTCs in pM1-staged prostate cancer [276]
**KRT7, KRT18, KRT19**	46.9%	Anti-KRT19 antibody HPA002465	KRT7, 18 and 19 expression in CTCs from ovarian, gastric and gastroesophageal cancer patients [277, 278] Used for therapy monitoring of advanced NSCLC and breast cancer[279]
**c-Met+/CD47+**	0.8-33.3%	Hu5F9-GA, ARG 197	CD44/c-Met/CD47 CTCs from breast cancer patients display metastatic potential [46] c-Met+/CD47+ CTCs as novel independent prognosticator of OS in luminal breast cancer [154, 236] c-Met as a capture antigen for CTCs and as a therapeutic target [237, 238, 280] CD47 expression on CTCs of colorectal cancer [239, 281]
**HER2**	7.9-35.9%	Herceptin, Pertuzumab, Lapatinib, Trastuzumab-mertansine (T-DM1)	HER2 expression on CTC of metastatic breast, non-small cell lung, gastric, gastrointestinal, ovarian cancer [12, 13, 36, 119, 178, 189, 282] Anti-HER2 therapy to address HER2-positive CTCs [283] HER2 is part of the signature of breast cancer CTCs competent for brain metastases [284]
**EGFR**	18-56%	Cetuximab, Afatinib, Erlotinib, Gefitinib, Panitumumab	EGFR expression on CTCs of colorectal, prostate, non-small cell lung, gastric, head and neck, and breast cancer [32, 36, 121, 210, 283, 285–288] Treatment resistance T790M EGFR mutation in CTCs of non-small cell lung cancer [289] Lapatinib treatment of metastatic breast cancer patients with EGFR-positive CTCs [290] EGFR is part of the signature of breast cancer CTCs competent for brain metastases [284]
**MUC1/16**	28.1-90%	ASI402	Expression of mucin 1 and 16 in CTCs from ovarian cancer patients [277]
**HPSE**	n.a.	PI-88	Breast cancer CTCs express heparanase [291] HER2/EGFR/HPSE/Notch1-positive breast cancer CTCs have brain metastastic potential [284]
**Androgen receptor**	16.3-18%	Bicalutamide, Flutamide	Nuclear expression of androgen receptor splice variant 7 protein in CTCs of metastatic castration-resistant prostate cancer is a treatment-specific biomarker that is associated with superior survival on taxane therapy over ARS-directed therapy [288, 292]
**Telomerase**	n.a.	Imetelstat	Telomerase activity on CTC of metastatic prostate cancer is a prognostic marker [293] Telomease-sensitive adenovirus as diagnostic and therapeutic tool against CTCs in various cancer [294, 295]
**Vimentin**	32.3%	Withaferin-A, Silibirin, Quercetin	Decrease OS of castration-resistant prostate cancer patients with vimentin/ki-67-positive CTCs [296]
**Ki-67**	20.8-45.1%	n.a.	Ki67 expression in CTCs of metastatic breast cancer [297, 298]
**M-30**	10-76.63%	M30 CytoDeath™ ELISA	Apoptosis-related fragment of keratin 8 generated by caspases Metastatic disease is associated with lower numbers of apoptotic CTCs [299]
**TWIST1**	n.a.	Curcumin, SFN, Quercetin, CADPE, Moscatilin, NAC, BMP7, Claudins	TWIST1 is expressed in CTCs of breast cancer patients along with further EMT and stem cell markers [269]
**uPAR**	n.a.	PAI-1, anti-uPAR antibody 10G7, WX-UK1, Mesupron	Expression of uPAR on subsets of CTCs in metastasized breast cancer [300] Co-amplification of HER2 and uPAR in CTCs of breast cancer [301]
**Markers for DTCs**			
**CD44**	33-100%	Pan-CD44 antibody H90	CD44 expression on most breast cancer DTCs [302]
**Survivin**	n.a.	ISIS23722, EM-1421	Survivin expression in bone marrow-resident DTCs in colorectal cancer [303]
**TWIST1**	31%	Curcumin, SFN, Quercetin, CADPE, Moscatilin, NAC, BMP7, Claudins	TWIST1 expression in bone marrow-resident DTCs in non-metastatic breast cancer [304, 305]
**uPAR**	58%	PAI-1, anti-uPAR antibody 10G7, WX-UK1, Mesupron	uPAR expression on DTCs of localized prostate cancer is an adverse prognostic marker [306]
**Thomsen-Friedenreich antigen**	98%	JAA-F11	Thomsen-Friedenreich antigen is expressed on bone marrow-resident breast cancer DTCs [307]
**HER2**	43%	Herceptin, Pertuzumab, Lapatinib, Trastuzumab-mertansine (T-DM1)	HER2 expression on DTCs in breast, ovarian and esophageal cancer [67, 282, 308] Gain of HER2 expression in esophageal cancer DTCs confers high risk of early death [67] HER2 expression on breast cancer DTCs as a prognostic marker for OS and PFS [309–311] 52% concordance of HER2 expression on primary tumor and DTCs in patients with early breast cancer [312]
**EGFR**	15-88%	Cetuximab, Afatinib, Erlotinib, Gefitinib, Panitumumab	EGFR expression on breast, colorectal and gastrointestinal cancer DTCs [313–315] Cancer-specific EGFRvIII mutant as a marker of breast cancer DTC [316] EGFR and FGF2 promote amplification of DTCs
**FGF2**	n.a.	Dovitinib, Pentraxin-3	
**NUAK1**	n.a.	WZ4003	Differential expression of NUAK1, PIN4, MALT1, and CDC25B in single prostate cancer DTC defines dormant subtypes .[317]
**PIN4**	n.a.	Anti-PIN4 antibody EPR10033	
**MALT1**	n.a.	EP603Y	
**CDC25B**	n.a.	Anti-CDC25B antibody S353	
**CEA**	0-84%	n.a.	CEA expression on breast, colorectal, and gastric cancer DTCs [315, 318, 319]
**EpCAM**	28.5-	Panorex, MT201, MT101, ING-1	Expression of EpCAM on non-small cell lung, breast, rectal, ovarian, prostate cancer DTCs [176, 317, 320–324] EpCAM-positive DTCs as therapeutic targets [323] Frequent loss of EpCAM expression on bone marrow-resident DTCs in esophageal cancer patients [10]

MUC: mucin; ALDH1: aldehyde dehydrogenase isoform 1;CD47: cluster of differentiation 47; EGFR: epidermal growth factor receptor; FDA: food and drug administration; EMT: epithelial-mesenchymal transition; mCRPC: metastatic castration resistant prostate cancer; HPSE; N-acetylcysteine, NAC; Caffeic acid 3,4-dihydroxy-phenethyl ester; uPAR: urokinase-type plasminogen activator receptor; MRD: minimal residual disease; CEA:carcinoembryonic antigen.

n.a.: not applicable.

Compared to CTCs, DTCs are further advanced cancer cells since they have already settled in distant organs such as the bone marrow or lymph nodes [22]. As such, DTCs might harbor valuable information concerning the metastatic potential of the disease and deserve therefore intensive analyses of associated antigens, which might represent therapeutic targets. For example, expression of EpCAM on DTCs of esophageal cancer patients was restricted compared to primary tumors, but correlated with lymph node involvement and remarkably poor outcome [10]. It must be noted however, that in comparison to CTCs, the detection of DTCs is more invasive, given the need for bone marrow puncture or surgery in case of lymphatic DTCs. For these reasons, longitudinal monitoring of DTCs is barely possible for routine clinical applications.

In the metastatic situation, CTC-based liquid biopsies might not only identify the right patients for more effective therapies but could help avoid futile treatment in de novo resistant cancers. Few initial experiments suggested that short-term *in vitro* expansion and testing of metastatic breast cancer CTCs permits prediction of the patient's response to drugs [23, 24]. But it is important to cautiously note that such short term CTC cultures are far from being validated clinical applications. Given the extremely few publications in this field and the numerous groups world-wide working on CTCs, it is obviously very difficult to establish reliable CTC culture systems. However, besides their enumeration, distinct molecular characteristics of CTCs were reported to predict recurrence and treatment response [25]. For example, more mesenchymal CTCs were associated with disease progression and treatment resistance in metastatic breast cancer [25], which is in line with the recently discovered function of EMT in chemoresistance in mouse models of metastatic breast and pancreatic cancer [26, 27]. Comparably, CTCs in prostate cancer can display androgen receptor (AR) expression and signaling transitions that could provide valuable information for second-line therapy with adequate inhibitors [28].

In the adjuvant, non-metastasized situation, enumeration of CTCs also has prognostic significance and indicates patients with risk for systemic progression [21, 29–31], with the potential to improve therapy and patient care. For instance, the increase of selected, more aggressive CTC phenotypes in patients clinically staged N_0_/M_0_ could represent a rationale for enhanced adjuvant treatment to prevent recurrence and metastases. As example, the presence of CTCs in locally advanced head and neck cancer patients after chemotherapy was predictive of poor survival except for oropharyngeal cancers, suggesting that CTCs have the potential to define patients who would profit from intensified therapy [32, 33]. Here, molecular staging could help to decide upon the timing to change or reinforce radiation and tailor systemic therapy regimens.

Pre-clinical and clinical trials including CTCs and DTCs for various clinical purposes are underway and address CTC enumeration as well as molecular characterization of a plethora of antigens (Table 1 and 2). For example, the potential of CTC numbers as a criterion for treatment decisions was addressed in the prospective randomized SWOG S0500 trial [34]. CTC counts were used to stratify metastatic breast cancer patients for continued standard therapy or for a treatment arm composed of an alternative chemotherapy. Unfortunately, the CTC-informed alternative chemotherapy had no beneficial effect on OS and PFS [34]. This negative result might be explained by selectivity issues of the CellSearch™ system [35], heterogeneity of CTCs [11, 35, 36], general resistance to chemotherapy, and eventually - and most likely - the choice of the alternative chemotherapy. Beyond that one first trial, enumeration of CTCs (also with CellSearch™ system) could be still predictive in a different clinical setting and several trials are currently ongoing to further investigate these aspects [15] (Table 2). First pilot phase results of the international EORTC 90091-10093 Treat CTC, phase 2 proof-of-concept trial (NCT01548677) have been disclosed very recently [37]. Here, CTCs are monitored in form of liquid biopsy in HER2-negative breast cancer patients (adjuvant and neo-adjuvant situation). Patients with detectable CTCs after radio-chemotherapy are stratified to an observational arm and a treatment arm, implementing the anti-HER2 antibody Trastuzumab [37]. So far, 11% of patients (*n* = 350) had detectable, treatment-resistant CTC after standard adjuvant treatment, out of which 26 patients (7.4%) have been randomized to either study arm. Results related to the efficacy of Trastuzumab to eradicate CTCs and clinical endpoints such as recurrence-free survival, invasive DFS, DFS and OS are expected two years after the last patients will have been randomized [37].

**Table 2:** Selection of ongoing trials related to CTCs of solid tumors. (According:https://clinicaltrials.gov/; assessment date:08/11/2016)

ClinicalTrials.gov Identifier	Title/study	No of patients	Time period	Primary endpoints	Cancer type
**CTCs as biomarkers or therapeutic targets**					
**NCT01548677**	Trastuzumab in HER2-negative Early Breast Cancer as Adjuvant Treatment for Circulating Tumor Cells (CTC) (“TREAT CTC” Trial)	2175	Apr 2013- Dec 2018	CTCs detection	Breast cancer
**NCT01619111**	DETECT III - A Multicenter, Randomized, Phase III Study to Compare Standard Therapy Alone Versus Standard Therapy Plus Lapatinib in Patients With Initially HER2-negative Metastatic Breast Cancer and HER2-positive Circulating Tumor Cells	120	Feb 2012-Mar 2018	CTC clearance rate	Breast cancer
**NCT01975142**	Validity of HER2-amplified Circulating Tumor Cells to Select Metastatic Breast Cancer Considered HER2-negative for Trastuzumab-emtansine (T-DM1) Treatment.	480	Oct 2013-Nov 2016	Tumor response rate to T-DM1 in patients with HER2 amplified CTCs	Breast cancer
**NCT01349842**	CirCe01 Study: Evaluation of the Use of Circulating Tumour Cells to Guide Chemotherapy From the 3rd Line of Chemotherapy for Metastatic Breast Cancer	568	Jan 2010-Jan 2018	OS	Breast cancer
**NCT00382018**	S0500 Treatment Decision Making Based on Blood Levels of Tumor Cells in Women With Metastatic Breast Cancer Receiving Chemotherapy	651	Oct 2006-May 2017	OS, PFS	Breast cancer
**Predictive, diagnostic and prognostic value of CTCs**					
**NCT02610764**	Pilot Study: Resectable Esophageal Adenocarcinoma and the Relevance of CTC (ESO-CTC)	20	Nov 2015-Dec.2017	Changes of CTC numbers	Esophageal cancer
**NCT02035813**	DETECT IV - A Prospective, Multicenter, Open-label, Phase II Study in Patients With HER2-negative Metastatic Breast Cancer and Persisting HER2-negative Circulating Tumor Cells (CTCs).	520	Jan 2014-Dec 2019	PFS	Breast cancer
**NCT01322893**	Enumeration and Molecular Characterization of Circulating Tumor Cells in Women With Metastatic Breast Cancer	150	Mar 2011-Dec 2016	CTC numbers	Breast cancer
**NCT02626039**	Characterization & Comparison of Drugable Mutations in Primary and Metastatic Tumors, CTCs and cfDNA in MBC patients (MIRROR)	40	Nov 2013-Dec 2016	Mutations and genomic alterations in primary tumor tissue and metastases	Breast cancer
**NCT02119559**	Assessment of Circulating Tumor Cells as an Early Predictive Marker of Response to a First Line Treatment Based on an Anti-Human Epidermal Growth Factor Receptor (HER), Cetuximab, in Patients With Inoperable Recurrent and/or Metastatic HNSCC.	115	Sep 2012-Mar 2018	Predictive value of CTCs on PFS	Head and neck squamous cell carcinoma
**NCT02554448**	Detection of CTCs in Stage III Rectal Cancer Patients Undergoing Neoadjuvant Therapy	80	Jan 2016-Dec 2016	CTC numbers	Rectal cancer
**NCT01596790**	Assessment by EPISPOT of Circulating Tumor Cells as an Early Predictive Marker of Response to Chemotherapy and Targeted Therapy in Patients With Metastatic Colorectal Cancer in First Line of Treatment	168	Apr 2012-Apr 2016	Predictive value of CTCs on PFS	Colorectal Cancer
**NCT01848015**	Prediction of Recurrence in Advanced Gastric Cancer After Radical Resection by Circulating Tumor Cells (CTCs)	200	Jun 2013-Jul 2016	CTC as predictive marker for recurrence	Gastric cancer
**NCT01625702**	Clinical Significance of Circulating Tumor Cells (CTCs) in Blood of Patients With Advanced/Metastatic Gastric Cancer	100	Jun 2012-Dec 2015	CTC as prognostic marker	Gastric cancer
**NCT02072616**	Detection of Circulating Tumor Cells for the Diagnostic of Pancreatic Adenocarcinoma	142	Sep 2014-Sep 2021	Sensitivity of CTCs as diagnostic marker	Pancreatic cancer
**NCT02451384**	Comparison of the Influences of Different Methods to Remove the Pancreatic Ductal Adenocarcinoma on the Detection of Circulating Tumor Cells	45	Jul 2015-Dec 2016	CTCs between the pre and post-operation in each study arm	Pancreatic ductal adenocarcinoma
**NCT02155426**	A Multicenter, Prospective, Observational Trial on the Prognostic and Dynamic Change of CTC Enumeration in Advanced NSCLC With 1st or 2nd Line Chemotherapy and Targeted Therapy	1200	Apr 2014-Dec 2016	Baseline CTC count	Non-small cell lung cancer
**NCT02407327**	Individualized Treatment of Patients With Advanced NSCLC: Potential Application for Circulating Tumor Cells (CTC) Molecular and Phenotypical Profiling (2012/52)	150	Dec 2013-Dec 2017	Percentage of CTC-positive patients and total CTC numbers	Non-small cell lung cancer
**NCT02500693**	Circulating Tumor Cells and Early Diagnosis of Lung Cancer in Patients With Chronic Obstructive Pulmonary Disease	600	Nov 2015-Dec 2019	CTC detection rate	Lung cancer
**NCT02372448**	Multicenter Validation of the Sensitivity of Theranostic ALK Rearrangement Detection by FISH Analysis and Prevalence of Escaping Mutations in Circulating Tumor Cells for the Non-invasive Management of Lung Cancer Patients	224	Jul 2014-Jul 2016	Sensitivity and specificity of the FISH technique in CTC assessment	Lung cancer
**NCT02666612**	Measurement and Characterization of Circulating Endothelial Cells or Circulating Tumor Cells in Adult Patients With Metastatic Cancer	1000	Aug 2008-Aug 2020		Metastatic cancer
**NCT01961713**	Circulating Tumor Cell Analysis in Patients With Localized Prostate Cancer Undergoing Prostatectomy	200	Apr 2010-Apr 2019		Prostate cancer

PFS: Progression Free Survival; OS: Overall Survival CEC: circulating endothelial cells; EPC: endothelial progenitors cells

While the discussed increasing trial activity testing CTC-based liquid biopsy in metastasized patients is encouraging, a comprehensive transfer to the adjuvant situation is missing. The major bottleneck here is the rarity of CTCs in the M0 situation and the low blood volume (< 10 mL) usually investigated, insufficient to reliably detect the few CTCs present [15, 16, 19, 20, 38]. A potential solution to overcome this problem might be the use of diagnostic leukapheresis (DLA), which enables density-based pre-enrichment from large blood volumes (liters) and thereby the screening of liters of blood for CTCs [39]. However, this approach must be validated in larger cohorts with respect to feasibility and prognostic value [15, 39].

Alternative to CTC/DTC based liquid biopsies, circulating tumor DNA (ctDNA) has been extensively investigated for diagnostic and prognostic purposes [40]. ctDNA is released into the circulation by tumor cells following apoptosis and necrosis, and represent a comparably simple tool for the analysis of systemic disease [41, 42]. ctDNA isolation can be performed from serum and blood plasma, requires less sophisticated technologies and is less cost intense. For instance, cancer personalized profiling by deep sequencing (CAPP-Seq) was applied for the case of non-small cell lung cancer and demonstrated the presence of ctDNA in 100% of stage II-IV patients and 50% of stage I patients [43]. ctDNA levels correlated with tumor burden and outperformed radiographic approaches with respect to treatment response assessment [43]. As such, ctDNA analysis might represent a complementary tool to the CTC analysis [44, 45]. Despite obvious advantages of simplicity for the isolation and analysis through standardized deep-sequencing methods, ctDNA clearly harbors several drawbacks compared to the enumeration and characterization of CTCs and DTCs. While ctDNA-based diagnostic is currently closer to clinical routine use, CTCs and DTCs allow the analyses at genomic, transcriptomic, and proteomic levels, whereas ctDNA analysis remains restricted to genomic alterations. Furthermore, CTCs and DTCs can be further studied *in vitro* for resistance traits [23, 24] and *in vivo* in animal models for their metastatic capacity [46]. Thereby, the gain of knowledge acquired through the analysis of CTCs and DTCs is incomparably more comprehensive. Thus, CTCs (and possibly DTCs) represent superior candidates for liquid biopsy since they have the potential to reflect disease progression and therapy response at multiple biological levels [25, 47–53]. However, substantial challenges remain in efficient enrichment, detection, and isolation of CTCs due to potential loss of capture antigens during EMT [11, 15, 16, 35, 54–57], in usage as liquid biopsy owing to the small volumes of peripheral blood currently analyzed [15, 19, 58], in genetic and molecular profiling to enhance our knowledge of the metastatic cascade [22, 59–69], and in therapeutic targeting at the earliest time points to attack the very cells possibly responsible for lethal metastases [16, 20, 57, 70–81].

## CTCS AND DTCS AS PRECURSORS OF METASTASES: HINTS AND EVIDENCE

**Figure 1:**
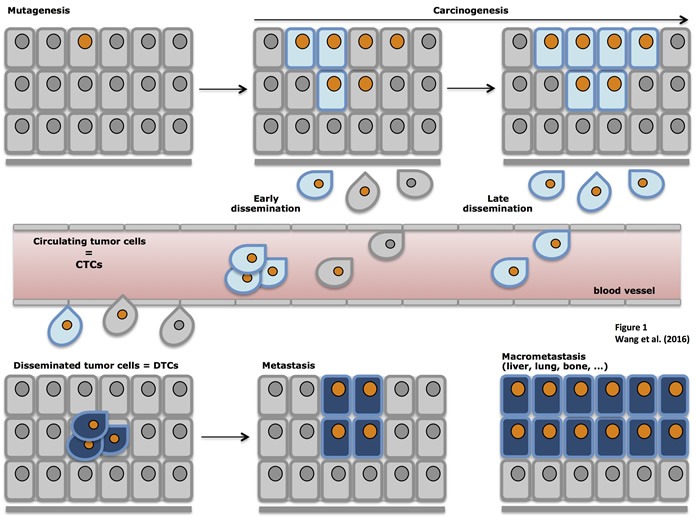
Schematic representation of tumor progression Primary carcinomas are induced through multiple mutations and the outgrowth of malignant cells *in situ*. Upon epithelial-to-mesenchymal transition (EMT) cells acquire migratory and invasive traits, detach from primary tumors and locally invade surrounding tissue. In a next step, locally invading cells gain access to blood or lymph vessels through intravasation and become circulating tumor cells (CTCs). After extravasation, CTCs settle in novel organs/sites and are termed disseminated tumor cells (DTCs), which can give rise to micro- and macrometastases in various organs.

So far, the true metastatic potential of CTCS remains largely unclear. The metastatic cascade is initiated by detachment from primary tumors, local invasion and intravasation into the blood. When the invasive cancer cells become blood-borne they are called CTCs. After extravasation at secondary sites, the cancer cells can settle and are then termed DTCs (Figure 1) [54, 70, 71, 82–84]. The actual time point of the metastatic spread remains a highly interesting question. Does the metastatic cascade represent a late process requiring dissemination of fully malignant cells from locally advanced tumors or is it the result of early spread of distinct cells that undergo co-evolutionary changes parallel to the primary tumor [22, 61, 69, 85]? Malignant cells that are less changed by evolutionary developments within the primary tumor but rather have to adapt “on site” in a new micro-environment might be more capable of metastases formation [63, 69, 86]. Whichever theory is eventually correct (most likely both scenarios can occur in cancer patients and can explain the different individual courses of disease), the general assumption is that the process of metastases formation is poorly efficient.

Data on a direct contribution of CTCs and DTCs to metastases formation remains scarce up to now, but the few available data indicate that CTCs and DTCs are indeed metastatic precursors and therefore direct targets for systemic therapy. In breast cancer patients, presence of DTCs at the time point of primary tumor diagnosis or following systemic treatment strongly correlated with metastasis at distant sites [84, 87]. Proof of a tumorigenic potential of DTCs came from cell lines generated from micrometastatic DTCs from lymph nodes of patients suffering from esophageal cancers, which generated tumors in immune-compromised SCID mice [88]. Direct evidence for a tumorigenic potential of CTCs came from metastatic breast cancer [46] and aggressive small cell lung cancer (SCLC) [89] that is characterized by early dissemination and utterly poor prognosis.

Seminal proof of a metastatic potential of circulating cells was recently published for luminal breast cancer, formally demonstrating the existence of metastasis-initiating cells (MICs) amongst CTCs [46]. Intrafemoral injection of a minimum of > 1,000 human CTCs into immune-compromised NSG mice induced the development of bone, lung and liver metastases with a latency time to disease of 6-12 months. Taking into account that values of > 5 CTCs per 7.5mL of blood showed prognostic correlation with poor survival of breast cancer, these patients would display an approximated total load of > 3,500 CTCs in circulation and theoretically surmount thresholds required for metastases formation in mouse models. Obviously these calculations are rough estimations and extrapolate numbers from animal models to human disease. In fact, only four out of 106 patients analyzed complied with the above mentioned requirements of > 1000 CTCs per injection, out of which CTCs from 3 patients actually generated metastases [46].

Metastatic potential is highly enriched in clusters of CTCs present in the blood of patients. In metastatic breast cancer, oligoclonal clusters are held together through plakoglobin-activated adhesion and harbor > 20-fold increased metastatic potential compared to single cell CTCs [90]. Hence, metastatic breast cancer CTCs comprise subpopulations with metastatic potential, however efficiency and frequency of MICs appear slight or can not be properly monitored with the current experimental tools.

Subcutaneous xenotransplantation of CTCs from distantly metastasized small-cell lung cancer patients into NOD-SCID-IL2-receptor gamma chain deficient (NSG) mice induced tumor formation. Four out of six samples of CTCs generated palpable tumors in a time range of 2.4-4.4 months and reflected the patient's response to platinum and etoposide [89]. Numbers of CTCs inoculated (20-1,625) correlated with the time to generate palpable tumors and > 400 CTCs per 7.5mL of blood were required for tumor formation in xenotransplants. Additionally, circulating tumor cell-derived xenografts (CDX) from SCLC also induced the formation of distant metastases in lungs and brains of mice, hence demonstrating a metastatic potential. Importantly, CTCs and CDXs from individual patients shared genomic alterations, but displayed intratumoral and especially intertumoral heterogeneity [89]. Such heterogeneity is clinically relevant given its impact on treatment, chemoresistance, dissemination and metastases formation in breast cancer [24, 25] and non-small cell lung cancer [91].

The abovementioned studies demonstrated formally that CTCs are tumorigenic and metastatic, confirming the assumed importance of CTCs in disease progression. Because CTCs can preserve morphological and genetic characteristics of primary tumors and faithfully recapitulate responses of donor patients to chemotherapeutic agents, they represent a means to develop precision medicine strategies based on routinely monitoring molecular features of CTCs. In this context, CDXs become a major tendency as an alternative to PDXs, especially when tumors were inaccessible or difficult to biopsy [92].

It appears sensible to consider targeting of CTCs as a reservoir for MICs, but formal proof of the existence and metastatic potential of MICs is required for additional tumor entities to fortify this concept. Targeting CTCs/DTCs as precursors of metastases might be further complicated by plasticity and substantial heterogeneity observed not only in primary tumors but also in systemic cancer cells [25, 89, 93–98]. Metastatic potential could either be inherent to subclones of cells present in the primary tumor and/or be acquired by subsets of cells through mutations, epigenetic and transcriptional modeling of gene expression profiles [62, 68, 85, 99, 100], even at very early disease stages [61]. From a therapeutic point of view, targeting of CTCs and DTCs should concentrate on subsets with (regained) proliferative capacity as targets of chemotherapy and adjuvant immune-therapy. Here, the actual presence of target antigens for therapeutic antibodies must be thoroughly evaluated. In a second approach, induction of exit of dormancy in order to achieve sensitization for chemo- and radiotherapy [101] and inhibition of the switch from dormancy to proliferation [102] are valuable approaches to inhibit the outgrowth of MICs [103–106]. In this respect, it is of interest that the microenvironment present in bone marrow contributes to the regulation of tumorigenic traits, either silencing tumor cells into dormancy or re-activating them to circulate and proliferate. In breast cancer, tumor dormancy can be observed even up to decades before the outgrowth of overt metastases. For example, perivascular endothelial cells induce dormancy of breast cancer cells through the release of thrombospondin 1, whereas sprouting neo-vasculature accelerates cancer cell growth [106]. Hence, understanding initiation and regulation of tumor dormancy is yet another level of complexity and probably the furthest away from clinical application.

## DIRECT ANALYSIS OF SYSTEMIC CANCER FOR EFFICIENT TREATMENT

Molecular targets for cancer such as HER2, EGFR, EpCAM, VEGF, amongst others, have been defined in primary tumors and, selectively, in systemic cancer cells (Table 1). However, most cancers show marked intra- and inter-patient heterogeneity due to evolution of different clones and evolutionary changes to adapt to novel microenvironments [96, 99, 107–110]. As a result, measurement of molecular targets in primary tumors is insufficient to predict efficacy of adjuvant therapies because expression patterns in primary tumors are not systematically conforming those of CTCs and DTCs [111, 112]. Despite a frequent resemblance of antigen profile between primary tumors and metastases [113, 114], differences in gene and protein expression occur [115–118]. Breast cancer metastatic cells have for example been shown to re-express E-cadherin and catenins as opposed to the cognate primary tumors [116]. As a result, antigen-positive primary tumors can give rise to antigen-negative CTCs and DTCs, and vice versa, or to the expression of mutated antigen variants as was shown for EGFR and HER2 [10, 11, 13, 25, 35, 119–121]. Hence, patients with antigen-positive primary tumors might remain unaffected by antibody therapy owing to a lack of antigen on CTCs and/or DTCs, while patients with antigen-negative primary tumors but antigen-positive CTCs and/or DTCs will not be quoted as eligible for therapy. Thus, analysis of molecular markers should be conducted in primary tumors and repeatedly in liquid biopsies to thoroughly support decisions on therapeutic approaches. Optimally, a panel of markers with associated therapeutic agents should be included in such analyses. Beyond that, unbiased molecular characterization of CTCs, DTCs and metastases at the genetic and protein level will help to find new targets for improved therapy of systemic cancer [14–16, 19, 21, 48, 51, 52, 54, 57, 58, 64, 67, 68, 71, 73, 85, 86, 109, 122–128]. However, it must be noted that the technical requirements for the application of comprehensive liquid biopsies in clinical routine, especially in the adjuvant situation with all its restrictions, are not yet achieved.

## CURRENT MOLECULAR TARGETS

A recent analysis of cell surface markers of metastatic breast cancer-derived MICs as described by Baccelli *et al.* revealed the expression of epithelial marker EpCAM, hyaluronic acid receptor CD44, integrin associated protein CD47 and hepatocyte growth factor receptor c-Met as a signature for MICs [46]. EpCAM is generally used as anchor protein to enrich CTCs in various approaches [47, 129], CD44 is a marker for cancer stem cells in numerous tumor entities [130] including breast cancer [131], and is involved in metastases formation [132, 133], while CD47 and c-Met had been linked to recurrence and an invasive program of tumor cells [134, 135]. The frequency of CD44/CD47/c-Met triple-positive EpCAM-expressing CTCs increased by almost two-fold following disease progression and numbers of triple-positive CTCs were associated with higher metastatic burden, whereas simple enumeration of EpCAM-positive CTCs was not [46]. Hence, it can be assumed that these markers of breast cancer MICs provide cells with signals required for metastases formation *in vivo* and thus constitute possible therapeutic targets. A role for these MIC markers in metastases formation is further suggested by their frequent expression and functions in cancer stem cells of various entities [130, 136–140].

In the following, the above mentioned markers as well as additional, classical molecular targets will be discussed in light of their expression and availability on systemic cancer cells.

### Epithelial cell adhesion molecule EpCAM

EpCAM is, to date, the antigen of choice for the enrichment of CTCs out of the blood of patients [141]. The US food and drug administration approved the CellSearch^TM^ system relies on capturing CTCs *via* EpCAM-specific antibodies, and subsequent detection of DAPI positive, cytokeratin positive and CD45 negative objects [47, 52, 129].

Although EpCAM has great value for the capturing of CTCs from the blood of patients, drawbacks relate to its long assumed continuous expression in all phases of tumor progression, including circulating tumor cells. This assumption was based on the alleged constant expression of the molecule in primary tumors and actually represented a best candidate approach to enrich malignant epithelial cells from blood at the time CellSearch^TM^ was developed. EpCAM displayed epithelial specificity as well as frequent and high expression in numerous carcinomas [142, 143]. However, it is nowadays clear that EpCAM is subject to dynamic changes in expression throughout tumor progression, including changes related to mesenchymal transitions [11]. EMT and its reversion MET emerged as major driving forces that underlie phenotypic changes during tumor progression [107–109, 144] (Figure 1). Despite the knowledge that the expression of typical epithelial markers involved in cell adhesion and proliferation such as E-cadherin [145, 146] are lost with the induction of EMT [147–149], the possibility of a dynamic expression of EpCAM surfaced only more recently [11]. In fact, EpCAM can be lost on CTCs of various entities [12, 35, 150–153] as well as on DTCs [10], and CTCs enumeration might necessitate an upwards revision, as reported recently [16, 35, 152, 154, 155]. Whereas 15% of metastatic lung cancer patients displayed ≥5 EpCAM-positive CTCs in 7.5mL of blood, the percentage raised to 41% when taking EpCAM-negative cells into account [150]. Down-regulation or even complete loss of EpCAM in CTCs and DTCs might not only reflect ongoing EMT in these cells. Indeed, EpCAM functions as a central molecule in signaling, migration, regulation of cell cycle progression and tumorigenicity [156–159]. Active loss of EpCAM cell surface expression through endocytosis was seen in cells initiating migration [10, 160, 161]. Further analyses revealed increased migration and invasion of EpCAM-negative/low cancer cells [10, 162], which was however contradictory to reports on increased migration and invasion in the presence of EpCAM [163–166]. EpCAM-positive/high cancer cells were characterized by increased tumorigenicity, enhanced proliferation and diminished sensitivity towards growth factor deprivation [139, 156, 158, 159, 165, 167, 168]. Oncogenic potential of EpCAM is initiated *via* regulated intra-membrane proteolysis that generates a signaling active intracellular domain termed EpICD, which increases transcription of cell cycle and pluripotency regulators [156, 158, 160, 169–172]. Ultimately, EpCAM was recognized as a membrane protein that is strongly overexpressed in cancer stem cells of all major carcinoma entities [136, 137]. A contribution of EpCAM to “cancer stemness” is further conceivable given its capacity to stimulate pluripotency of embryonic stem cells [171, 173].

Thus, EpCAM emerged as a switch between traits of epithelial and mesenchymal cells. Interestingly, EpCAM-positive DTCs of esophageal cancer patients strongly associated with lymph node metastases and poor OS, but represented a minority in these patients, with approximately two-thirds bearing EpCAM-negative DTCs [10]. MICs defined in breast cancer patients expressed EpCAM strongly [46], so that EpCAM-positive CTCs constitute therapeutic targets. However, EMT switches were observed in primary tumors of breast cancer patients and even more so in CTCs [25]. Appearance of mesenchymal CTCs (EMT-CTCs) in patients correlated with disease progression and a resistance towards chemotherapy [25, 26]. Similarly, EMT-CTCs correlated with poorer OS in a small cohort of HNSCC patients [174], which might be explained by different capacities of epithelial and mesenchymal cancer stem cells in HNSCC [162].

**Figure 2:**
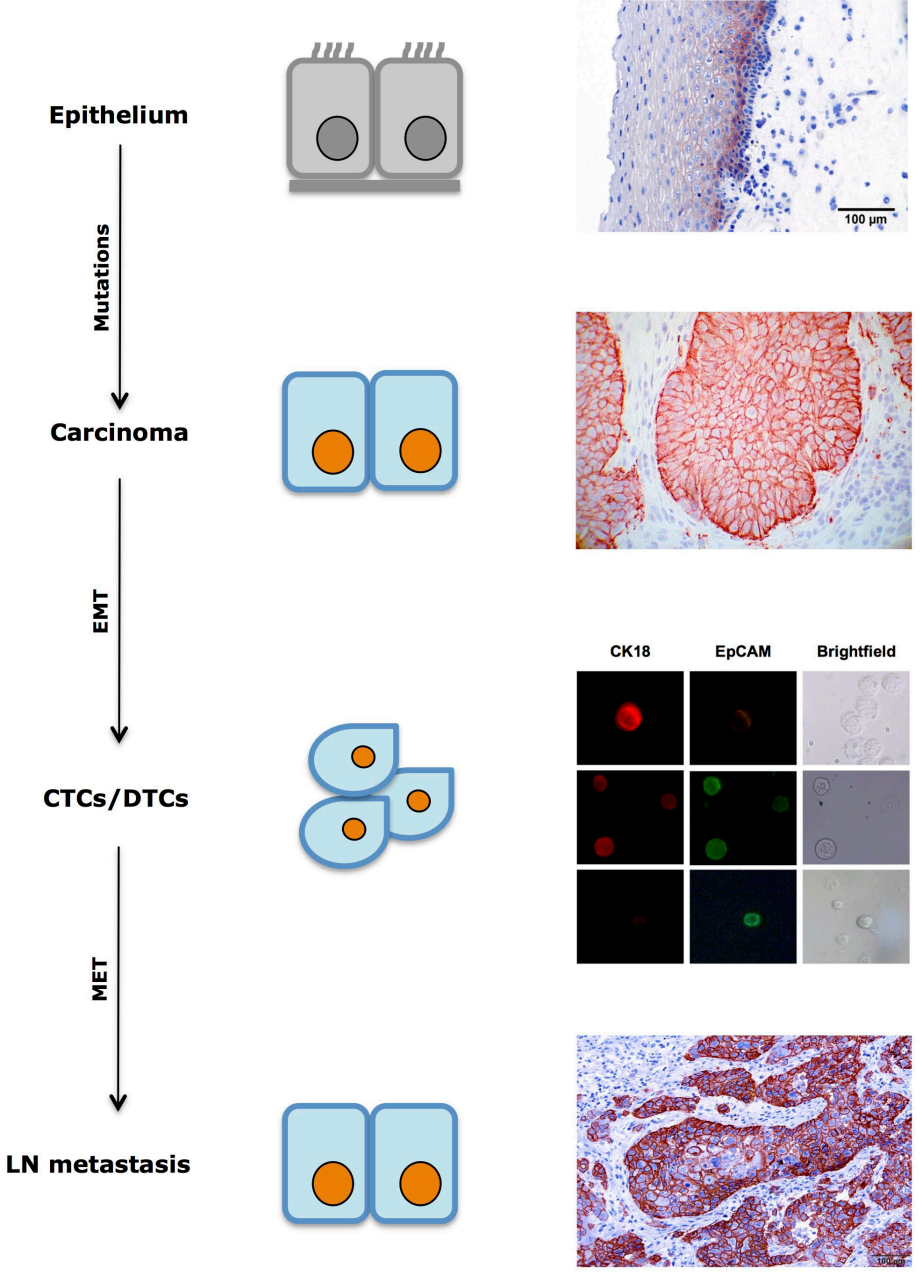
Dynamic expression of EpCAM in tumor progression EpCAM expression in normal mucosa is commonly restricted to cells of the suprabasal layers. During tumor formation through sequential mutations, EpCAM expression is frequently increased in cells of primary carcinomas. Circulating and disseminated tumor cells (CTCs/DTCs) display mixed expression patterns with retained or lost expression of EpCAM. Macrometastases is often characterized by strong expression of EpCAM, which is similar to the corresponding primary tumor. DTC immunofluorescence pictures displaying EpCAM status were taken with permission from [10].

Hence, EpCAM expression on primary tumor cells but also on CTCs might activate proliferation and tumor initiation at distant sites, and is a novel parameter, whose measurement might represent a surrogate for differing phenotypic states of cancer cells (Figure 2). In fact, EpCAM expression is dynamic and not steady as it was long assumed. In this respect, antigen-independent isolation of CTCs and DTCs becomes highly relevant in order to assess and study varying phenotypes of these cells in tumor progression, recurrence, metastases formation and treatment responses. Various recent CTC isolation and/or enrichment technologies have taken this notion into consideration and isolate CTCs through size and filtration separation [39, 175] or upon depletion of hematopoietic cells and assessment of cellular ploidy, as well as tumor biomarker expression [176, 177]. The later technique termed with iFISH combines the determination of polyploid tumor cells using chromosome enumeration probes with phenotypic immunofluorescence detection of markers of choice. Diversified subsets of CTCs or DTCs may possess distinct clinical significance in terms of drug resistance, cancer metastases and disease relapse [178].

Knowledge of EpCAM expression on CTCs and DTCs in the bone marrow could reveal of clinical and therapeutic importance, since existing monoclonal and recombinant antibodies (Panorex, MT201, MT101, ING-1) might experience a revival for the systemic targeting of tumorigenic CTCs and DTCs. Furthermore, small molecule inhibitors of EpCAM signaling addressing its cleavage could be envisaged in combinatorial therapies. Last but not least, determination of the epithelial *versus* mesenchymal status of CTCs and DTCs might represent a surrogate marker for therapy response and recurrence [25], which could be repeatedly assessed in peripheral blood in clinical routine.

## EPIDERMAL GROWTH FACTOR RECEPTOR 2 HER2

HER2 has become a central therapeutic target. Treatment with monoclonal antibodies or small molecules is currently a routine intervention for metastatic breast cancer patients expressing high levels of HER2 in primary tumor cells as measured upon the HercepTest™. HER2 is a receptor tyrosine kinase involved in regulation of cell proliferation and apoptosis *via* MAP-kinases, PI3/AKT and the mTOR pathway [179–184]. Opsonization of HER2-positive cells and functional inhibition of HER2 with therapeutic antibodies and small molecule inhibitors proved beneficial for node-negative and -positive as well as metastatic breast cancer patients [9, 185, 186]. HER2^high^ patients treated with Trastuzumab displayed a 12% increase in OS and a 33% reduction of the risk of death [186]. Owing to the longest history and most comprehensive knowledge [187], the impact of HER2 expression on CTCs with respect to disease outcome, as well as a benefit from anti-HER2 therapy for patients with HER2-positive CTCs were assessed. The prognostic value for the presence of CTCs with respect to OS was confirmed and stratification according to HER2 expression on CTCs was performed. Cut-off was set at > 30% of CTCs expressing HER2, which clearly correlated with response to treatment. Patients undergoing anti-HER2 treatment and bearing HER2-postive CTCs had significantly prolonged progression-free survival (8.8 *versus* 2.5 months) [188]. Furthermore, anti-HER2 treatment was efficient since patients bearing HER2-positive CTCs but left untreated had a very poor progression-free disease (1.5 *versus* 8.8 months) [188].

Potential benefit of targeting HER2-positive CTCs in patients is further addressed in the DETECT III study (NCT01619111). In this ongoing multi-center, randomized phase III study, metastatic breast cancer patients with initially HER2-negative primary tumors but HER2-positive CTCs are treated with standard therapy alone or standard therapy combined with Lapatinib treatment (anti-HER2/EGFR inhibitor). 711 out of 1123 HER2-negative patients enrolled in this study had measurable CTCs counts after EpCAM-mediated enrichment, and 134 patients had at least one HER2-positive CTCs in 7.5mL blood. This represents a percentage of discordance of primary tumor *versus* CTCs of 18.8%. Other research groups similarly reported on such discordance in expression profiles [12–14, 189]. Stratification of patients into subgroups demonstrated a strong and significant association of HER2-positive CTCs with hormone receptor-positive and lobular breast cancer. Assessment of the efficacy of Lapatinib treatment in addition to standard care is ongoing and highly anticipated.

Hence, anti-HER2 therapy, which is already in clinical routine for breast cancer patients, clearly demonstrated the power of molecular analysis and targeting of CTCs in the control of metastatic disease and is very encouraging.

### Epidermal growth factor receptor EGFR

EGFR is, similarly to HER2, a major target for targeted therapies *via* monoclonal antibodies and inhibitors [190], which belongs to the same receptor tyrosine kinase family [191–194]. In fact, EGFR is the founding member of this family of signaling receptors, which was discovered in 1978 [195]. EGFR signaling is broad and comprises differential activation modes through ligand induced phosphorylation and interaction with a multitude of intracellular pathways such as MAP-kinases, phospholipase C, phosphatidyl-inositol-3 phosphate kinase, small GTPases, and JAK/STATs [193, 196, 197]. Thereby, EGFR activation stimulates proliferation, migration, angiogenesis, differentiation, survival, cancer formation and progression [198, 199]. Besides classical signaling *via* phosphorylation-induced activation of downstream targets, EGFR was demonstrated to translocate into the nucleus and to activate transcription through association with target gene promoters [200–203]. Additionally, EGFR is subject to proteolytic cleavage at the membrane by members of the metalloproteinase and/or rhomboid protease family to generate an intracellular domain (ICD), the actual function of which remains undescribed [204–206].

Since anti-EGFR antibodies are part of late stage therapies, the status of EGFR-positive CTCs was assessed with the purpose to measure therapy responses to Cetuximab and to inquire a potential use of EGFR therapeutic antibodies for the eradication of CTCs [207–209]. In colorectal cancer patients, great intra- and inter-patient heterogeneity was observed at the level of EGFR expression and mutation status, which might explain differences in treatment responses [207]. Although intra-patient variance represents an issue, the actual expression of EGFR on CTCs consolidates the strategy of CTCs targeting through biological and small molecule inhibitors already available on the market. Current research though focuses on the detection of EGFR mutations in CTCs and ctDNA, as surrogate markers for monitoring purposes rather than stratification means for subsequent anti-EGFR therapies. These efforts have peaked in the launch of a specific test of EGFR mutation in ctDNA called Selector™. Additional studies reporting on steady expression levels of EGFR in breast cancer patient-derived CTCs [210], as well as an eradication of EGFR-positive and -negative CTCs following gefitinib treatment [211], further support the concept of antigen-specific targeting of CTCs. However, remaining CTCs in these breast cancer patients revealed negative for EGFR, which pinpoints at possible escape mechanisms that could be addressed through the use of multiple targeted treatments. Furthermore, radiotherapy reportedly increased numbers of EGFR-positive CTCs in locally advanced head and neck squamous cell carcinomas (HNSCC), which could be counteracted upon treatment with anti-EGFR antibody Cetuximab [32]. EGFR was associated with an EMT phenotype of non-metastatic breast cancer patients' CTCs, which co-expressed markers of mesenchymal cells such as vimentin and slug [212]. Hence, although somewhat unexpected, EGFR might be a positive regulator of EMT processes observed in subsets of CTCs, which are selected upon radiotherapy. However, numbers of HNSCC patients enrolled in CTC enumeration and EGFR evaluation was comparably small (*n* = 31) and further validation in larger cohorts is necessary.

Comparably to HER2, anti-EGFR antibodies and small molecule inhibitors are approved for clinical use for colorectal, head and neck squamous cell carcinomas (HNSCC) and non-small cell lung cancer. Both, HER2 and EGFR are therefore interesting targets to therapeutically address systemic disease that have already proven beneficial for cancer patients. Even more so, a combination of HER2- and EGFR-specific drugs appears valid since HER2 signaling emerged as one major route of resistance to Cetuximab, suggesting that Trastuzumab or equivalents could help overcoming resistance [213].

### Hyaluronic acid receptor CD44

CD44 in fact designates a family of more than 20 differing transmembrane proteins that are generated from the single *CD44* gene through extensive alternative splicing of 10 out of 20 exons, as well as post-translational modifications [214–216]. CD44 has multiple functions in adhesion to extracellular matrix, cytokines and growth factors presentation, migration and differentiation, cell and nuclear signaling [217–223]. Early on, expression of splice variants of CD44 such as CD44v6 was shown to stimulate metastases formation and was in the focus of cancer research [132, 133, 224, 225]. Further interest arose with the description of CD44 as a marker for cancer stem cells in various carcinoma entities including breast [131], colon [226], hepatocellular carcinomas [227], head and neck [228], lung and pancreatic cancers [229, 230]. Reasons for this recurrent expression of CD44 in tumor initiating cells of various malignancies including hematopoietic and epithelial cancers have been reviewed in depth and relate to the various roles mentioned above [130]. Eventually, CD44 must be considered as a signaling platform, which not only activates cell adhesion and migration through binding of ECM components, but also on proliferation, apoptosis, angiogenesis, differentiation and regulation of stemness through the activation of multiple pathways such as Wnt/ß-catenin, NF-κB, Src and PKC kinases, Rho GTPases [130, 138, 231]. As such, CD44 enables cells to react and respond to cues from the microenvironment, inducing a stem cell phenotype including the expression of stemness factors and the regulation of traits of metastatic cells [220, 221].

In their seminal work on MICs in breast cancer, Baccelli *et al.* provided the first translation of markers of cancer stem cells to a subpopulation of metastases-inducing CTCs, thus providing a MICs signature [46]. They combined the function of CD44 in metastases formation with its strong and frequent expression on CSCs to demonstrate for the first time the presence of CD44 on MICs. Thereby, CD44 became a potential target candidate for the eradication of MICs upon adjuvant therapies comprised of CD44-specific antibodies. Approaches to target acute myeloid leukemia cells using the monoclonal pan-CD44 antibody H90 proved very efficient [232], and might give a basis for future application in the eradication of MICs. However, given the potential of CD44-specific antibodies to target antigen-positive hematopoietic cells, knowledge of splice variants preferentially expressed on CTCs, and especially on MICs, would help designing therapeutics with an effectiveness more restricted to CTCs. In this respect, the expression of a sialofucosylated glycoform of CD44 termed HCELL for hematopoietic cell E/L-selectin ligand on tumor cells is of great interest. HCELL is a major ligand for both selectin subtypes, which allows the interaction of tumor cells with endothelium, leukocytes and platelets and might thus trigger intra- and extravasation of CTCs in and out of vessels during tumor progression [233, 234]. Therapeutic blockage of HCELL on CTCs, given its expression, would represent an elegant way to prevent dissemination and metastases formation.

In homology to EpCAM, HER2, and EGFR, all attempts to target CD44 on CTCs will depend on thorough knowledge of expression profiles. Thus, future clinical studies should optimally implement measurements of CD44 expression on CTCs.

### Hepatocyte growth factor receptor c-Met and Integrin-associated protein CD47

cMet and CD47 are emerging markers of importance owing to their capacity to foster migration and invasion [135] and control cells of the innate immune system [235], respectively. Luminal breast cancer patients harboring cMet/CD47-positive CTCs were at high risk of metastatic spread. Accordingly, double-positive CTCs displayed substantial ability to develop metastases in mouse models [78, 236]. The CellSearch^TM^ platform was modified to enrich for c-Met-positive CTCs, which were rare according to this study and might restrain the use of inhibitory monoclonal antibodies and inhibitors that are currently in clinical testing [237, 238] (http://meetinglibrary.asco.org/content/140112-158). Besides MICs in breast cancer, CD47 was strongly expressed on CTCs from colorectal cancer patients and might act as antagonist of innate immune cells during circulation [239, 240]. Hence, CD47 and c-Met are of great interest for therapeutic targeting of systemic cancer, but clearly require more in-depth analysis of expression and function on CTCs to warrant therapeutic addressing.

### Programmed cell death protein 1 PD-1 and its ligand PD-L1

PD1 and PD-L1 is a receptor-ligand pair of membrane proteins expressed on immune cells (T, B, macrophages, natural killer and myeloid cells), endothelial and epithelial cells [241]. Activation of PD-1/PD-L1 signaling results in immune suppression through inhibition of ZAP70 and protein kinase C variants in T cells [242–244]. PD-L1 is increased in carcinoma cells of numerous entities, and, as such, enables tumor cells to dampen activated T cell responses, thereby initiating cancer immune evasion [241, 245, 246]. Accordingly, expression of components of the PD-1/PD-L1 axis, also termed PD pathway, correlated with poor prognosis and survival of carcinoma patients [247]. Therapeutic inhibition of the PD pathway displayed great potential to reactivate immune cells and induce long-lasting remissions [244, 248, 249]. Thus, PD treatment represents one of the most promising cancer therapies of the moment [241, 250–252], with checkpoint inhibitors comprising both, PD-1 and PD-L1 targeting therapeutic antibodies in clinical trials (see Tables 1–4 in [252]).

Importantly, PD-L1 expression was demonstrated on CTCs in various carcinoma entities including breast [253, 254], oral [255], colorectal and prostate [256], lung cancer (http://meeting.ascopubs.org/cgi/content/abstract/34/15_suppl/e23036). Interestingly, Satelli *et al.* used the cell-surface vimentin (CSV)-specific antibody to isolate EMT-CTCs and demonstrated differing sub-cellular localization of PD-L1. Nuclear localization of PD-L1 in EMT-CTCs was associated with poor prognosis of colorectal and prostate cancer patients [256]. For the case of ovarian cancer, expression of PD-L1 in primary tumors correlated with peritoneal dissemination and the generation of ascites, suggesting a role for PD-L1 in the inhibition of cytotoxic T cells and dissemination, which was confirmed in mouse models [257].

Hence, PD-L1 expression on CTCs has once more dual potential for the identification of patients likely to respond to PD treatment in the context of liquid biopsies and as therapeutic target to reactivate the immune system towards systemic cancer cells.

## CONCLUSIONS

**Figure 3:**
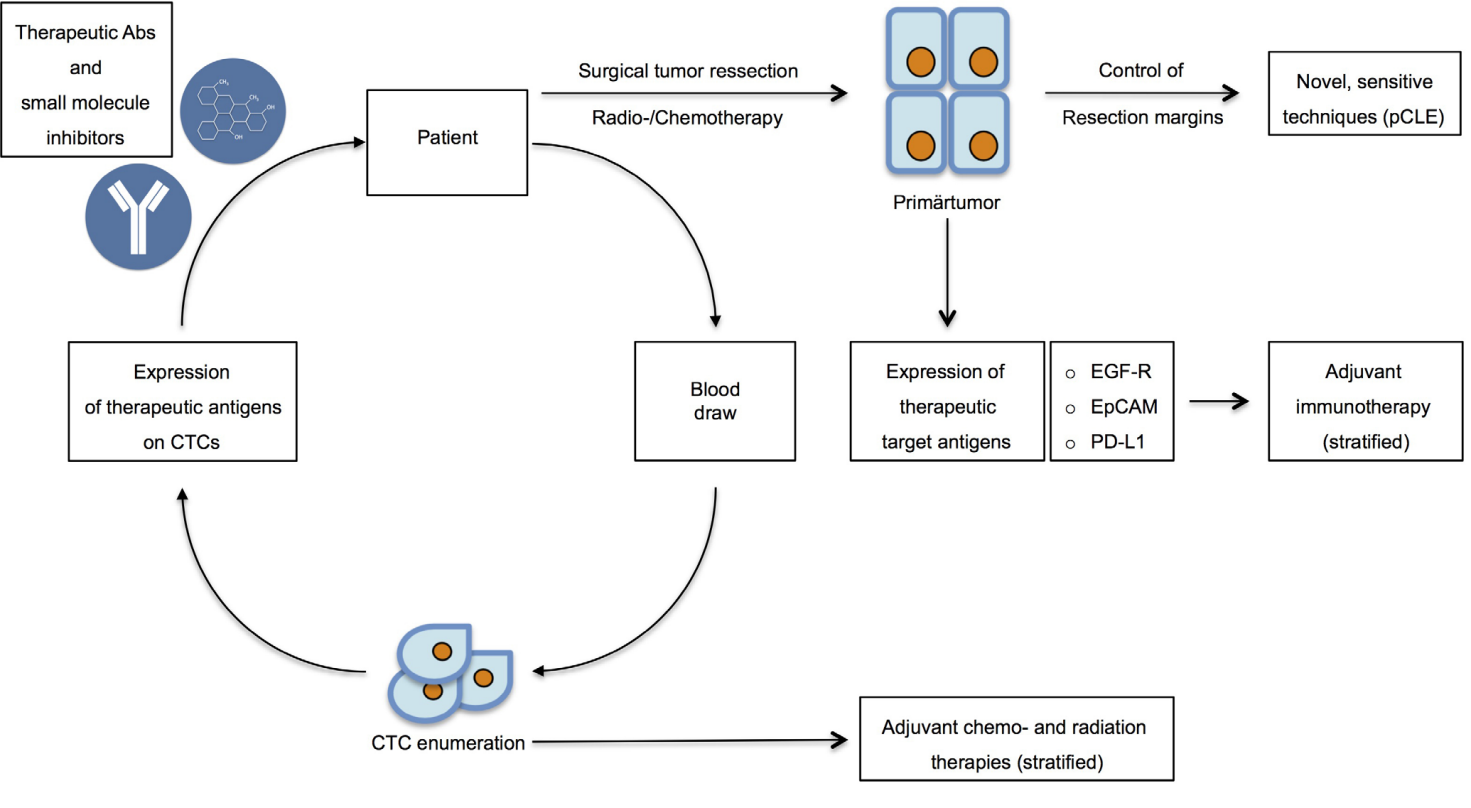
Therapeutic options in targeting CTCs and DTCs After initial diagnosis, patients eligible for operation undergo surgical resection of the primary tumors in combination with chemo- and radiation therapy. Resection margins should be controlled through novel, sensitive techniques including probe-based confocal laser endomicroscopy (pCLE) to assure complete withdrawal of tumors. Routinely, the expression of therapeutic target antigens such as *e.g.* EGFR, EpCAM and PD-L1 should be assessed in order to improve adjuvant therapy through adequate stratification. Simultaneously, blood draws will serve to assess CTC numbers and to perform molecular characterization of the expression of therapeutic antigens. CTC enumeration will be implemented into decisions concerning adjuvant chemo- and radiation therapies. Molecular profiling of CTCs will allow for the determination of the application of novel therapeutic antibodies and small molecule inhibitors.

Metastasis is the major thread for cancer patients and, despite progress in the era of molecular therapy, remains incurable in most cases. Surgical options for the removal of metastases are limited and systemic treatment has been so far rather ineffective. Research on molecular mechanisms involved in metastases formation suggested a central role of circulating and disseminated tumor cells. The majority of evidence supports the notion that CTC-based molecular analysis has the potential to provide real-time and non-invasive surrogates to enable better diagnostics, prognostication, and prediction. Subsets of CTCs expressing cell surface markers EpCAM, CD44, CD47 and c-Met were capable of initiating metastases in animal models [46] and hence, these seminal findings might pave the way for novel strategies in cancer therapy because potential targets of therapy, both cellular and molecular, become apparent (Figure 3). It must however be noted that a formal proof of the metastatic capacity of CTCs subpopulations has to the best of our knowledge only been given for metastatic breast cancer and small cell lung cancer, and is thus lacking for other entities.

Currently, cancer patients are eligible for adjuvant therapies targeting cell surface antigens such as HER2 and EGFR, primarily in late stages of disease, when metastases have already developed or tumors relapsed. Clinical interventions might profit from monitoring CTCs and the repeated analyses of the expression of molecular targets such as HER2, EGFR, EpCAM and PD-L1 on CTCs during the course of targeted therapies. Based on these analyses, early application of therapeutic agents targeting markers on MICs could be considered and clinically addressed. A basic requirement is to have reliable assays at hand that deliver such data. In view of the plethora of promising available assays, it is therefore of utmost importance to standardize and validate such assays. This is currently addressed for lung cancer and a breast cancer subtype by a large EU/IMI consortium (www.cancer-id.eu). Similar initiatives must be extended to other cancer types and, especially, to the adjuvant situation.
